# Metal-on-metal metaphyseal and ceramic-on-ceramic femoral neck arthroplasty: the impact on clinical results, oxidative stress and concentration of metal ions in serum and blood

**DOI:** 10.1007/s00590-023-03540-y

**Published:** 2023-04-05

**Authors:** Tomasz Stołtny, Michał Dobrakowski, Aleksander Augustyn, Sławomir Kasperczyk, Dominika Rokicka, Rafał Skowroński, Krzysztof Strojek, Bogdan Koczy

**Affiliations:** 1 District Hospital of Orthopaedics and Trauma Surgery in Piekary Śląskie. Bytomska, St. 62, 41-940 Piekary Śląskie, Poland; 2 Department of Biochemistry, Faculty of Medical Sciences in Zabrze, Medical University of Silesia in Katowice, Jordana St. 19, 41-808 Zabrze, Poland; 3 Department of Internal Diseases, Diabetology, and Cardiometabolic Diseases, School of Medicine With the Division of Dentistry in Zabrze, Silesian Centre for Heart Diseases in Zabrze, Medical University of Silesia in Katowice, M. Curie-Skłodowskiej 9, 41-800 Zabrze, Poland; 4 “ALFA” Orthopaedics and Traumatology Center Ul. Ogrodniczki, 51 15-763 Białystok, Poland; 5 Solskiego St. 46 42-609, Tarnowskie Góry, Poland

**Keywords:** Metaphyseal hip arthroplasty, Femoral neck hip arthroplasty, Metal ions, Metal-on-metal articulation, Ceramic-on-ceramic articulation, Reactive oxygen species, Oxidative stress

## Abstract

**Purpose:**

Growing number of hip arthroplasty in Poland performed with the use of metaphyseal stems results from the decreasing age of patients qualified for procedures and is consistent with the corresponding trends in European countries. To this day, a significant population functions after undergoing hip replacement using metal-on-metal implant. This study was aimed at the assessment of the variability of the oxidative system, as well as the concentrations of chromium and cobalt ions in serum and blood and their potential impact on postoperative clinical status.

**Material and methods:**

The analysis included 58 men. The first group—operated using J&J DePuy ASR metal-on-metal implant with metaphyseal stem Proxima^Tm^. Second group—operated using K-Implant SPIRON® femoral neck prosthesis in full ceramic articulation. Selected parameters of oxidative stress and the antioxidant system as well as the concentration of metal ions in blood were determined twice. Each patient underwent two clinical evaluations using acclaimed physical examination scale systems.

**Results:**

In the first group, significantly higher concentrations of Cr (*p* = 0.028) and Co (*p* = 0.002) were demonstrated compared to the group of femoral neck arthroplasty. The mean concentrations of Cr and Co, 10.45 and 9.26 μg/l, respectively, were higher in patients operated bilaterally. In the ASR group, greater pain intensity in the operated hip and higher indicators of oxidative stress were found.

**Conclusions:**

Metal-on-metal articulation of the hip significantly increases the concentration of Cr and Co in blood, induces oxidative stress and modifies function of the antioxidant system and generates greater pain in the operated hip.

## Introduction

Hip osteoarthritis has been named a leading cause of disability worldwide, and its prevalence continues to increase due to the ageing population and the global obesity epidemic. In the early stages of the disease therapeutic exercise, broadly defined as bodily movement prescribed to correct impairment, improve musculoskeletal function or maintain a state of well-being [1].

Total hip replacement remains the leading surgical procedure in radical treatment of hip osteoarthritis. The decreasing age of patients with primary osteoarthritis of the hip joint qualified for elective arthroplasty affects the growing number of such treatment procedures performed in developed countries [2].

At the end of the second decade of the twenty-first century in Germany Klug et al., analyzing the type of primary hip arthroplasty and the method of their placement, set the direction for the development of hip arthroplasty. The obtained results showed an increasing number of performed cementless classic and metaphyseal arthroplasties, along with the decreasing frequency of implanting resurfacing and cemented endoprostheses [3].

In femoral neck arthroplasty, a high and selective line of the femoral neck osteotomy with the complete preservation of the Adams' arch determines the maximum bone saving in the proximal end of the femur. Femoral neck arthroplasty systems were invented for younger, active patients, in whom the fact of early implant placement resulted in an increased probability of repeated revision operations in the future. By placing the stem in the femoral neck, they promote physiological load transfer and reduce the stress-shielding phenomenon, leading to the preservation of greater bone mass compared to the use of classic cementless stems [4].

The increasing use of hip arthroplasty has significantly contributed to reducing pain, improving mobility, and improving the quality of life of operated patients. Second-generation metal-on-metal hip replacement systems were developed at the beginning of the twenty-first century as an alternative to the widely used metal-on-polyethylene implants. However, the emerging reports on the increased concentration of metal ions in the blood in patients with the metal-on-metal configuration and increased pain, hypersensitivity reactions or even carcinogenic potential prompted the consideration of the rationale for their further use. Another solution was sought—ceramic-on-ceramic articulation [5].

It is believed that the basis of the toxic effects of chromium and cobalt on the human body is their ability to generate reactive oxygen species [6]. The imbalance between their production and removal is known as oxidative stress, which leads to the development of many disease states. The most important intracellular sources of reactive oxygen species include the respiratory chain, the nicotinamide adenine dinucleotide phosphate (NADPH) oxidase, and peroxisomes [7]. The first line of defense against reactive oxygen species is superoxide dismutase (SOD) [8]. This enzyme catalyzes the decomposition of the superoxide radical anion to hydrogen peroxide, which is then broken down by catalase and glutathione peroxidase [9]. The activity of the last enzyme depends on the availability of reduced glutathione. The compound acts as the main non-enzymatic thiol antioxidant that provides reducing equivalents to other antioxidants and scavenges reactive oxygen species [10].

The aim of the study was the double assessment of the variability of oxidative system parameters and the concentration of chromium and cobalt ions in blood and plasma as well as postoperative status evaluation based on the recognized clinical scales in the population of men with coxarthrosis after primary metal-on-metal metaphyseal and ceramic-on-ceramic femoral neck arthroplasty.

## Material and methods

The analysis included 58 male patients of the District Hospital of Orthopaedics and Trauma Surgery in Piekary Śląskie with the diagnosis of osteoarthritis of the hip joint and operated using metaphyseal or femoral neck arthroplasty. The patients were divided into two research groups:

- Group I: 34 patients after unilateral and 3 patients after bilateral metal-on-metal hip arthroplasty J&J DePuy ASR with the Proxima^Tm^ metaphyseal stem.
- Group II: 21 patients who underwent K-Implant SPIRON® femoral neck arthroplasty using the ceramic-on-ceramic Biolox Delta—II.

### Description of the implants used in patients qualified for the study

In the first group, metal-on-metal articulation with the J&J DePuy ASR system with the Proxima^Tm^ stem was used. It consists of a press-fit chrome–cobalt–molybdenum cup and a head made of the above-mentioned metal alloys, combined with a metaphyseal stem made of a titanium alloy coated on its entire surface with hydroxyapatite, which was placed uncemented [11].

In the second group, femoral neck arthroplasty of the hip joint was performed with the use of cementless components consisting of CORIN pressfit cup and the K-Implant SPIRON® femoral neck stem which used BIOLOX Delta II ceramic-on-ceramic articulation.

It is manufactured from bioactive titanium alloying with a calcium phosphate coating. Sizes available include 18, 20, 22, 24 mm in diameter and 50, 55, 60 and 70 mm in length [12].

### Research methodology

Patients from each study group were assessed at two follow-up visits. In group I, the first visit took place about 5 years after surgery (mean 58.9 months), in group II—about 3 years after surgery (mean 34 months). The second visit was on average 6 months after the first. Besides clinical evaluation, blood was collected at each visit for oxidative stress parameters along with cobalt (Co) and chromium (Cr) concentrations.

Inclusion criteria for the study were: men 30–60 years old, intra-operatively assessed normal cancellous bone density of femoral neck, normal geometry (CCD angle 125°–135°), idiopathic degenerative disease of the hip, aseptic necrosis of femoral head and an patients’ informed consent.

Exclusion criteria were: women (due to the specifics of the ward), previous femoral neck fracture treated using operative or non-operative methods, distorted geometry of femoral neck and patients’ not giving their consent.

Quantity difference between both studied groups should be considered as a limitation of this study.

Biochemical determinations were performed in the Department of Biochemistry. Faculty of Medical Sciences in Zabrze. Medical University of Silesia in Katowice, in the Laboratory of District Hospital of Orthopaedics and Trauma Surgery in Piekary Śląskie and in the Toxicology Laboratory of the EkoProfMed Medical Center in Miasteczko Śląskie.

The determinations of cobalt (Co) and chromium (Cr) concentrations were made using the Atomic Absorption Spectrometer by Thermo Fisher Scientific, Great Britain, model ICE 3400 with a GFS35Z graphite furnace, with an automatic sample feeder, with background correction based on the transverse Zeeman effect and deuterium correction on the QuadLine lamp. The chromium concentration was determined according to Huang by measurement at a wavelength of 357.9 nm and cobalt according to Schwingel Ribeiro at a wavelength of 242.5 nm. Metal concentrations are presented in μg/l [13, 14].

Ceruloplasmin (CER) concentration in serum was determined according to Richterich [15] with *p*-phenyldiamine reaction. Sulfhydryl group (SH) concentration was determined as described by Koster et al. [16]. The results were shown in μmol/l.

The level of malondialdehyde (MDA), a lipid peroxidation product, was evaluated fluorometrically in serum and erythrocytes according to Ohkawa et al. [17] with minor modifications. Concentrations were expressed as µmol/l serum and in µmol/gHb erythrocytes. The concentration of lipofuscin (LPS) was measured according to the method of Tsuchida et al. [18]. The results were expressed as relative units (RU) per gram of hemoglobin in erythrocytes and in RU/l in serum (the fluorescence of a 0.1 mg/ml solution of quinidine sulfate in sulfuric acid is equal to 100 RU).

The activities of superoxide dismutase (SOD) were measured in serum and erythrocytes according to Oyanagiu [19]. The enzymatic activity of SOD was given in nitric units. The activity of SOD is equal to 1 nitric unit (NU) when it inhibits nitric ion production by 50%. The activities of SOD were normalized to milligrams of Hb (NU/mg Hb). Catalase (CAT) activity in erythrocytes was measured by the kinetic method of Johansson and Håkan Borg [20].

The activity of CAT was expressed as kU/g Hb. The activity of glutathione reductase (GR) in erythrocytes was measured according to Richterich [15]. The activity was expressed as *μ*moles of NADPH utilized per minute per g hemoglobin in erythrocytes (IU/g Hb).

Glutathione peroxidase (GPX) activity in erythrocytes was measured by the kinetic method of Paglia and Valentine [21]. The activity of GPX was expressed as micromoles of NADPH oxidized per minute per g hemoglobin in erythrocytes (IU/g Hb).

Clinical evaluation of patients was performed based on the physical examination and using the WOMAC-HIP, HHS and the SF-12 quality of life scale. WOMAC-HIP is a recognized multi-dimensional disability assessment and self-assessment tool for osteoarthritis. The best result is 0 points, the worst 96 points [22]. The HHS scale assesses pain, function, deformity and joint mobility. A maximum of 100 points can be obtained [23]. The SF-12 scale is a proven and widely accepted tool for assessing the quality of life in terms of physical and mental health. The physical health category includes the following subscales: physical functioning, the role of physical constraints, physical pain, and general health. The mental health category includes: vitality, social functioning, the role of emotional constraints and mental health [24]. To assess the perception of pain, the VAS scale was used, in which the measurements taken from the starting point (left end) of the scale to the mark drawn by the patient are recorded in centimeters and interpreted as their pain. The values can be used to track pain progression in a patient or to compare pain between patients with similar conditions [25].

In the physical evaluation, the length of the lower limbs was assessed twice (possible shortening or lengthening of the operated limb) and the efficiency of the hip abduction apparatus in the Trendelenburg test also was evaluated. Using a goniometer, the following mobility measurements were taken: flexion, abduction, adduction, inward and outward rotational movements of the hip joint. To test the muscle strength of the quadriceps muscle of the thigh, a 5-point scale according to Lovette was used.

### Statistical analysis

For statistical analysis MS Excel 2019 and Statistica 10.0 PL Software were used. The mean and standard deviation (SD) were determined for continuous variables. Shapiro–Wilk’s test was used to verify normality. Levene’s test was used to verify the homogeneity of variances. Statistical comparisons between the groups were made using a *t*-test and a *t*-test with a separate variance or Mann–Whitney *U* test. Changes at the significance level of *p* ≤ 0.05 were considered statistically significant.

## Results

The general clinical characteristics of the studied groups are presented in Table 1.

**Table 1:** Clinical characteristics of the studied population. (data presented as mean ± SD or %)

Parameter	SPIRON K-Implant group (*n* = 21)		J&J DePuy ASR group (*n* = 34)		*p*-value
	Mean/%	SD	Mean/%	SD	
Ischemic heart disease	0.0%		0.0%		1.000
Hypertension	33%		24%		0.437
Diabetes	0.0%		2.9%		0.437
BMI (kg/m^2^)	28.6	3.55	28.4	3.30	0.858
Age at the time of surgery (years)	44.1	9.0	47.9	10.1	0.160
Age at the time of inspection (years)	46.9	9.43	54.6	10.01	**0.006**
Head size diameter (mm)	38.1	3.71	47.3	1.70	** < 0.001**
Cup size diameter (mm)	55.2	2.79	54.0	2.15	0.070
Local reaction (pseudotumor)	0.0%		0.0%		1.000

Bold values indicate that the statistical *p* value is below 0.05 (*p* < 0.05)

The values of the range of motion in all planes of the operated hip joint as well as the muscle strength are as shown in Table 2 and comparable in the patients of both groups. Shortening of the operated limb compared to the opposite side in the J&J DePuy ASR/Proxima group (*p* = 0.05) is a noticeable tendency.

**Table 2:** Assessment of the range of mobility, muscle strength and clinical condition of the assessed hip joint (data presented as mean ± SD)

Parameter	SPIRON K-Implant group		J&J DePuy ASR group		*p*-value
	Mean	SD	Mean	SD	
Flexion	109.0	13.7	110.6	13.0	0.678
Abduction	40.5	6.7	38.2	6.7	0.235
External rotation	29.3	10.0	27.4	7.3	0.413
Internal rotation	16.8	6.8	17.8	8.8	0.655
Adduction	29.5	6.7	31.2	6.4	0.365
HHS Sum	85.2	15.9	81.6	20.1	0.495
Symmetry/Asymmetry	0.36	1.15	−0.24	1.05	0.050
Trendelenburg symptom	4.8%		0.0%		0.206
Lovett muscular strength	4.86	0.36	4.88	0.41	0.817

The obtained data presented in Table 3 show a greater feeling of pain from the operated hip among patients in the J&J DePuy ASR/Proxima group compared to the SPIRON K-Implant group using VAS scales (*p* = 0.049, *p* = 0.043). Both study groups did not differ statistically significantly in terms of results on the clinical status assessment scales (WOMAC—hip, SF-12) with a significant improvement in relation to the condition before surgery in both cases.

**Table 3:** Clinical assessment, pain and mood assessment in the studied groups of patients (data presented as mean ± SD)

Parameter	1 visit					2 visit—after 6 months				
	SPIRON K-Implant group		J&J DePuy ASR group		*p*-value	SPIRON K-Implant group		J&J DePuy ASR group		*p*-value
	Mean	SD	Mean	SD		Mean	SD	Mean	SD	
WOMAC stiffness	0.86	1.46	1.29	1.90	0.371	0.90	1.41	1.62	1.72	0.117
WOMAC pain	2.14	2.54	3.24	4.02	0.271	2.24	3.32	3.35	4.04	0.293
WOMAC daily act	10.1	11.1	11.1	15.0	0.810	9.2	12.9	13.9	14.8	0.238
WOMAC-sum	13.1	14.4	15.6	19.9	0.626	12.4	17.2	18.9	19.8	0.219
VAS 1–10	1.24	1.37	1.88	2.35	0.259	1.14	1.56	2.15	2.28	**0.043**
VAS%	0.07	0.09	0.18	0.23	**0.049**	0.10	0.15	0.17	0.23	0.208
Number of painkillers	0.43	0.51	0.21	0.41	0.080	0.38	0.50	0.18	0.39	0.094
Hamilton-Score	2.10	1.22	1.68	1.09	0.193	2.14	1.42	2.09	1.46	0.892
SF12-physical health	15.7	3.35	14.9	3.70	0.421	15.9	3.17	14.9	3.72	0.323
SF12-mental health	20.6	3.76	22.4	4.70	0.152	21.7	3.34	22.1	4.10	0.693
SF-12-total	36.3	6.5	37.3	8.0	0.645	37.5	5.9	37.0	7.4	0.772

Bold values indicate that the statistical *p* value is below 0.05 (*p* < 0.05)

As shown in Table 4. the following parameters were observed in the J&J DePuy ASR group compared to the SPIRON K-Implant group: a higher intensity of oxidative stress in the form of a lower concentration of sulfhydryl (SH) groups by 16% (1 visit), a higher concentration of ceruloplasmin (2 visit), a higher concentration of malondialdehyde (MDA) in erythrocytes by 29% and a higher concentration of lipofuscin (LPS) in the serum by 72% (2 visit) and in erythrocytes by 76 and 56% (1 and 2 visit). GPx activity was decreased by 20 and 9% (1 and 2 visit), and catalase increased by 25%, SOD activity on subsequent visits showed different trends in activity changes.

**Table 4:** Oxidative stress and antioxidants enzymes in the groups of patients at particular stages of the study (data presented as mean ± SD)

	1 visit					2 visit—after 6 months				
Parameter	SPIRON K-Implant group		J&J DePuy ASR group		*p*-value	SPIRON K-Implant group		J&J DePuy ASR group		*p*-value
	Mean	SD	Mean	SD		Mean	SD	Mean	SD	
SH (μmol/l)—serum	242	45.2	203	48.2	**0.004**	273	71.3	262	73.6	0.588
CER (mg/dl)—serum	39.3	7.09	38.6	8.47	0.769	27.7	10.19	39.3	12.30	**0.001**
SOD (NU/ml)—serum	19.5	3.35	17.3	2.31	**0.006**	18.6	4.28	20.4	3.68	**0.037**
LPS (RF)—serum	887	230	824	187	0.275	445	332	764	245	** < 0.001**
MDA (μmol/l)—serum	2.30	0.93	2.12	1.12	0.527	3.30	1.39	2.54	0.77	0.076
GR (IU/g Hb)—erythrocytes	7.99	1.64	7.42	1.94	0.263	7.96	1.55	9.05	1.96	**0.035**
CAT (kU/g Hb)—erythrocytes	473	125.7	591	77.7	** < 0.001**	490	89.8	529	74.8	0.087
SOD (NU/mgHb)—erythrocytes	164	31.7	154	16.00	0.116	151	16.4	157	25.87	0.342
GPx (IU/gHb)—erythrocytes	59.2	12.31	47.2	9.98	** < 0.001**	67.6	9.03	61.4	10.17	**0.006**
LPS (RF/gHb)—erythrocytes	816	381	1440	413	** < 0.001**	867	327	1355	822	**0.012**
MDA (μmol/gHb)—erythrocytes	0.41	0.08	0.39	0.06	0.314	0.29	0.08	0.37	0.10	**0.002**

Bold values indicate that the statistical *p* value is below 0.05 (*p* < 0.05)

Cr and Co concentrations were more than 6–sevenfold higher in the J&J DePuy ASR group (*p* < 0.05) as presented in Table 5.

**Table 5:** Concentrations of studied metal ions in blood in the groups of patients at particular stages of the study (data presented as mean ± SD)

	1 visit						2 visit—after 6 months					
Parameter	SPIRON K-Implant group		J&J DePuy ASR group		Change %	*p*-value	SPIRON K-Implant group		J&J DePuy ASR group		Change %	*p*-value
	Mean	SD	Mean	SD			Mean	SD	Mean	SD		
Cr concentration (μg/l)	1.25	1.53	9.47	16.70	659%	**0.029**	1.40	2.75	11.43	20.19	717%	**0.028**
Co concentration (μg/l)	0.98	1.05	8.29	10.08	749%	**0.002**	1.19	1.77	10.23	17.95	762%	**0.026**
Co/Cr ratio	1.21	1.59	1.39	1.02	15%	0.493	1.37	0.88	1.16	0.95	−15%	0.405

Bold values indicate that the statistical *p* value is below 0.05 (*p* < 0.05)

As shown in Table 6, positive correlations were found between the elevated concentrations of chromium and cobalt ions and the increased feeling of pain (VAS 1–10, VAS%), as well as CAT concentrations in erythrocytes. Positive correlation between elevated Cr ions and LPS concentration in serum was noted, whereas positive correlations between elevated Cr, Co ions and LPS concentration in erythrocytes was found. On the other hand, negative correlations were shown between elevated concentrations of CR, Co, and GPx concentration in erythrocytes.

**Table 6:** Correlation between study metals and VAS and oxidative stress in ASR group (Spearman R *p* < 0.05) NS—non-significant

Parameter	Cr (μg/l)	Co (μg/l)
VAS 1–10	0.21	0.20
VAS %	0.20	0.21
SOD (NU/ml)—serum	NS	NS
LPS (RF)—serum	0.22	NS
GR (IU/g Hb)—erythrocytes	NS	NS
CAT (kU/g Hb)—erythrocytes	0.27	0.25
GPx (IU/gHb)—erythrocytes	− 0.26	− 0.28
LPS (RF/gHb)—erythrocytes	0.29	0.28

## Discussion

In our study, the concentrations of Co and Cr in the group of patients undergoing arthroplasty with the J&J DePuy ASR Proxima^Tm^ implant were higher than the concentrations of these ions in patients in the study by Maurer-Ertl et al., in which analogous implants were used [26]. Both in the group of our patients and in the group of patients of Maurer-Ertl et al. ion concentrations did not influence further therapeutic decisions.

A similar conclusion was made by Hjorth et al. Despite the concentration of Co and Cr ions higher than 7 μg/l, the authors did not observe negative consequences of the increased concentration of metal ions and thus did not qualify the patients for revision surgery [27]. Also during our study we did not qualify patients for revision surgery, despite Co and Cr concentrations above 7 µg/l. We did not observe any lysis around the implant, pseudotumors or aseptic loosening of endoprostheses.

Increased concentrations of Co and Cr may correlate with larger diameters of heads in metal-on-metal articulation and a longer period of time between implantation of the endoprosthesis and the ion concentration measurement [28].

Large size implants were used in our study, namely the mean diameter of the acetabulum was 54 mm, while the modular metal head was on average 47.3 mm. They were closely related to the high concentrations of the assessed Co and Cr ions. In over 5-year follow-up period, despite the above-established relationship, there was no need for revision surgery in the operated patients.

The correlation, similar to the one presented in our study, concerning the size of the used implants and the concentration of metal ions, however, present in the female sex, was previously established by Lainiala et al. [29].

White et al. compared the numerical groups of patients who used the metal-on-polyethylene configuration or the ceramic-on-polyethylene configuration. At that time, they found significantly higher concentrations of Co ions in patients with a metal head diameter of 36 mm compared to a head diameter of 32 mm. Moreover, almost a quarter (7 out of 30 patients) in the group with metal heads reported mild pain in the area of the operated joint [30].

The concentration of chromium and cobalt ions in whole blood and in the synovial fluid may be used to evaluate patients after metal-on-metal hip arthroplasty reporting the presence of a painful hip.

Taunton in his study notes the high correlation of cobalt concentration with unwanted local tissue reactions compared to other tests. He also reports that with a Co to Cr ion concentration ratio higher than 1.4 in patients after hip arthroplasty, the sensitivity in detecting adverse effects on the surrounding tissues is as high as 95% [31].

The main goal of hip arthroplasty is to reduce pain, increase mobility within the joint, improve the patient's vital functions in terms of movement, and improve the patient's quality of life. When analyzing the impact of the type of hip implant on the abovementioned elements of the patient's life, we used the WOMAC-HIP, HHS and SF-12 clinical evaluation scales. We did not find an advantage for any group in terms of patient functioning, joint mobility and deformity. The severity of pain was significantly greater in the group of patients undergoing metal-on-metal arthroplasty. This observation probably results from the toxic effect of the released metal ions from the used implants.

The main components of the alloys in endoprostheses are chromium and cobalt. [32]. As a result of corrosion and friction of the implant surface, metal micro- and nanoparticles are released into the surrounding tissues, which may result in adverse local tissue reaction (ALTR), which is one of the main causes of surgery failure. Additionally, the released metal ions can enter the bloodstream and thus trigger systemic reactions in the body [5].

Chromium is a metal that is widely used in industry, for example, in the manufacture of pigments, chrome plating, leather tanning, stainless steel production and welding [33]. This element disturbs, among others, the functions of the respiratory, urogenital and digestive systems, and also causes dermatological diseases. Cobalt is used, among others, in the process of polishing diamonds, metal alloys, porcelain, chemical and pharmaceutical industries [32]. Cobalt may additionally induce respiratory and nervous disorders, cardiomyopathy and hearing impairment [34].

It has been proven that chromium and cobalt—as redox-active metals—are a source of reactive oxygen species, e.g., catalyzing the Fenton reaction [34, 35]. As a result, they induce oxidative stress, which leads, among others, to the damage of cell membranes in the process of lipid peroxidation. One of the products of this process is malondialdehyde (MDA) [33]. In the present study, the concentration of MDA in erythrocytes was significantly higher in the J&J group than in the SPIRON group at the second visit (*p* = 0.002). Parameters determining the intensity of oxidative stress also include lipofuscin [36]. Its concentration in erythrocytes in the J&J group was significantly higher than in the SPIRON group, both during the first (*p* =  < 0.001) and the second visit (*p* = 0.012). A similar difference was observed for the concentration of this compound in the serum during the second visit. Additionally, positive correlations were revealed between the concentration of lipofuscin and the concentrations of the tested metals. These observations suggest that the release of metal ions from endoprostheses may induce oxidative stress. Its severity is also evidenced by the lower concentration of thiol groups in the J&J group than in the SPIRON group, recorded during the first visit.

It has been proven that chromium and cobalt can directly bind to proteins, including antioxidant enzymes, and also compete with other metals for binding sites in active centers, which may lead to impairment of their activity [35]. These reports explain the lower glutathione peroxidase (GP*x*) activities observed in the J&J group than in the SPIRON group during both the first and second visits. Additionally, negative correlations were observed between GP*x* activity and chromium and cobalt concentrations. Positive correlations were found between the concentrations of these metals and the activity of catalase, which was higher in the J&J group than in the SPIRON group during the first visit. Similarly, the activity of glutathione reductase (GR) and the antioxidant properties of ceruloplasmin was higher in the J&J group than in the SPIRON group at the second visit. The increase in the activity of antioxidant enzymes or antioxidant concentrations under conditions of increased oxidative stress should be interpreted with the organism's adaptation, in line with the theory of hormesis [37]. Hence, it should be assumed that the activity of antioxidant enzymes may depend on many factors, constituting the resultant of the action of opposing mechanisms at the time of measurement. On the one hand, their activity may decrease as a result of the direct toxic effect of a given element or compound, and on the other hand, it may increase as a result of activation of protective mechanisms in response to increased oxidative stress. In this way, the obtained values of superoxide dismutase activity should be interpreted, which in the J&J group during the first and second visits were, respectively, lower and higher than in the SPIRON group.

The observed differences in this study between the values of the studied parameters during the first and second visits should be interpreted in the context of the biokinetics of metals released from prostheses. The half-life of 80–90% of the amount of cobalt in the blood is several days, while the remainder is excreted over the years. In the case of chromium, 95% of the amount circulating in the blood is excreted within hours. The remainder has a half-life of 15–30 days [32]. Hence, it should be assumed that the concentrations of these metals in the blood and their biological effects are the resultant of the dynamic process of their release from the implant and of their excretion, which are in equilibrium with each other. As these metals are released largely by the friction mechanism of the surface of the endoprosthesis, their release over a given period of time can be significantly influenced by physical activity, which varies in individual people over time depending on many different factors. Hence, the 6-month interval between the measurements carried out in the framework of the study, which significantly exceeds the half-lives of chromium and cobalt in the blood, justifies the observed differences between their concentrations and the differences between the concentrations/activities of the remaining related parameters.

In addition, the interpretation of the obtained values of the parameters of oxidative stress and the functions of the antioxidant system is hampered by the fact that the average age of people in the J&J group was higher than in the SPIRON group, which should be considered a limitation of the study.

The relationship between the higher concentration of metal ions and hip pain in patients after hip arthroplasty in the metal-on-metal configuration was demonstrated by Smeekes et al. The above results correlate with those obtained in our study. In addition, they also relate to the use of large-sized heads over an average follow-up period of 30 months. Moreover, based on the SF36, HOOS and OHS questionnaires and the radiological assessment, clinically better results were obtained in the group of patients with cobalt concentration below 5 μg/l [38].

In turn, the Chinese authors in current reports indicate the need for revision surgery in almost ¼ (24.3%) of operated patients in a 10-year follow-up period after cementless hip arthroplasty using the J&J DePuy ASR system. A longer period of observation of the ASR XL system correlates with a significant increase in the concentrations of chromium and cobalt ions in the blood and deteriorating clinical results in the commonly used quality of life scales available for evaluation and is associated with the need for revision surgery [39].

Due to various reports and our obtained results regarding the effectiveness and safety of metal-on-metal implants, further observation and monitoring of patients with this type of articulation are recommended. Hip arthroplasty using the J&J DePuy ASR system entails the necessity of further monitoring of the concentrations of chromium and cobalt ions. The increasing concentration of Co and Cr ions and the ratio of these concentrations > 1.4 can be used to select patients requiring revision surgery.

## Conclusions

1. J&J DePuy ASR metal on metal arthroplasty with the Proxima^Tm^ metaphyseal stem increases the concentration of chromium and cobalt ions in the blood compared to femoral neck arthroplasty with ceramic-on-ceramic articulation.
2. Significantly elevated concentrations of Co and Cr ions in the blood of patients undergoing J&J DePuy ASR—Proxima^Tm^ metal-on-metal configuration correlate with the increased pain sensation in the operated hip, induce oxidative stress and modify the function of the antioxidant system.

## Data Availability

The authors confirm that the data supporting the findings of this study are available within the article. Raw data concerning the patients are being held in District Hospital of Orthopaedics and Trauma Surgery in Piekary Śląskie. Bytomska St. 62, 41–940 Piekary Śląskie, Poland.
